# The Need for Modernization of Biosecurity in the Post-COVID World

**DOI:** 10.1128/msphere.00025-22

**Published:** 2022-02-28

**Authors:** Diane DiEuliis, James Giordano

**Affiliations:** a National Defense University, Washington, DC, USA; b Georgetown University, Washington, DC, USA

**Keywords:** COVID-19, biosecurity, gain of function, modernization

## Abstract

At present, there are two hypotheses about the emergence of SARS-CoV-2; the first is that it was due to a naturally occurring zoonotic jump, and the second contends that it spread due to an accidental dispersion of a laboratory-acquired infection in Wuhan, China. While the pandemic’s actual origins remain occluded, it is useful to examine the latter possibility as a paradigm for evaluating biosecurity policy in the post-COVID world. While the pandemic may not have emerged from a research lab, this is possible with research on dangerous pathogens and prompts questions for biosecurity. How might biosecurity protections for such research be modernized while still enabling important, necessary public health research that utilizes dual-use or gain-of-function capabilities? As the world takes urgent action to mitigate shortcomings in the response to COVID-19, such questions and their potential solutions are vital to inform and direct future life science and technology endeavors.

## COMMENTARY

COVID is now regarded as one of the greatest challenges to global public health since the influenza pandemic of 1918. One consistency in the years between then and now has been the commitment to a robust and ubiquitous public health infrastructure for response. However, much has changed in the past 100 years, not only societal changes that affect transportation, communication, economics, and the lifestyles of individuals but also tremendous advances in science and technology. We have described how the response to the COVID-19 pandemic could and should be “modernized” through use of these advances, including modernization of the national stockpile and supply chains for a more successful response to future pandemic threats (1–3).

However, we posit that such advances in today’s science and technology also pose dual-use risks that warrant ongoing, vigilant biosafety and biosecurity consideration. Applications of synthetic biology for research discovery in the life sciences and for the creation of biomanufactured commodities have increased tremendously over the past decade (4). Thus, just as public health preparedness and response platforms require modernization to adopt advanced technologies for better preparedness and response, so too do biosecurity policies in light of such dual-use capabilities. Gene editing technologies and an expanding convergence between biotechnology and information technology have enabled precision manipulation of biology, which creates opportunities for harm only wished for during Cold War bioweapons programs. It is provocative that concerns about such dual-use capabilities may have spurred theories that SARS CoV-2 resulted from bioweapons-type experimentation. There is ample evidence to confirm that this was not the case (5), but there are two existing hypotheses of how SARS CoV-2 emerged, and there are indications in support of both. Early reports noted the likelihood of a naturally occurring zoonotic jump (6), whereas somewhat later in the pandemic it was speculated that lapses in biosafety and biosecurity could have caused a laboratory leak or laboratory-acquired infection (LAI) (7, 8). While the best analyses, including those of the U.S. intelligence community, remain inconclusive as to the specific source of SARS CoV-2 (9), we suggest that the possibility of a laboratory leak warrants policy scrutiny.

## THE UTILITY AND VALUE OF “WHAT IF” SCENARIOS

The possibility that a bioengineered SARS-CoV-2 emerged by accident from a virology laboratory provides a model for reviewing biosecurity controls. Namely, how were biosafety and biosecurity policies applied? Are there gaps or weaknesses in those policies? If a biosecurity leak had occurred, what would have been an appropriate response on behalf of the international community? These questions are not merely hypothetical but establish key issues for multinational efforts proposing large-scale and resource-intensive enhancements to collaboratively strengthen global health security and response capabilities (10).

The importance of strong global public health capabilities cannot be overemphasized and have consistently been proposed since the anthrax attacks of 2001 (11). The United States’ National Biodefense Strategy advocates this approach and supersedes previous federal policies to include both naturally occurring and perpetrated events. Moreover, careful analysis by the National Academies (12) has confirmed that public health infrastructure still provides a fundamental deterrent against the harmful use of emerging technologies such as synthetic biology.

However, if public health response capabilities are to be modernized, synthetic biology and gain-of-function (GOF) research are critical tools through which preparedness and response can be advanced. However, the benefits from these tools must be balanced with recognition, and mitigation, of their dual-use risks (13). For example, in 2018 researchers created an extinct horsepox virus *de novo* (14), causing concern about the dual-use implications of the synthetic biology tools used and prompting examination of the rigor of prevailing biosafety, biosecurity, and other governance controls (15). Similar methods of inquiry could be used to investigate how gaps in laboratory procedure could have resulted in COVID19, including

• extent of adherence to global biosafety and biosecurity compliance of high containment laboratories, including the appropriate containment of engineered strains, or synthetic DNA constructs that have pathogen properties;
• engagement of appropriate risk-benefit assessments of GOF research;
• role and applicability of international organizations, treaties, and/or governance in maintaining oversight of national and private laboratory activities.

In the case of SARS-CoV-2, it is notable that the Wuhan Institute of Virology (WIV) was one of several high-containment laboratories built in China after the first SARS outbreak in 2002. A benevolent view of Chinese national intent affords that GOF experiments performed there were expressly in the interest of preparing for and preventing a future SARS outbreak; indeed, the United States maintained collaborations with, and funding of, the WIV for this research (16), details of which have been recently analyzed (17). The United States’ policy requires that foreign-funded laboratories performing select agent research be subject to the requirements of the Select Agent Program (SAP), which includes site inspections and personnel background checks. Several laboratory-acquired infections had already occurred in biosafety laboratories across Asia (18), underscoring that at a minimum, the United States should stringently screen funded international laboratories to ensure their adherence to the highest level of biosafety and biosecurity required domestically. Outside the United States, the Organization for Economic Cooperation and Development (OECD) promotes guidelines for best practices and responsibility for outcomes in laboratories that engage research on potentially dangerous pathogens. To wit, we ask how rigorously were LAI guidelines and/or SAP requirements followed and enforced in the WIV?

We also query whether there is appropriate oversight and governance of transparency and reporting of high-containment laboratory incidents that have potentially harmful outcomes. A summary by the U.S. State Department enumerates a number of concerns regarding the biosafety and transparency of the WIV in particular (19). While member countries are expected to report outbreaks of international concern to the World Health Organization (WHO), how do these reporting requirements and protocols relate to LAIs? The State department notes that COVID-19 emerged several weeks before it was reported and that failure to note this emergence significantly hobbled early efforts to contain its spread.

In the United States, such incident reporting is a crucially important method of evaluating the effectiveness of containment measures (20), and both the Centers for Disease Control and Prevention (CDC) and the American Biological Safety Association maintain searchable databases of relevant data on potentially pandemic pathogens to develop best practices to mitigate risk. Although infectious spread is a direct risk, there is also the indirect risk of GOF materials being accessed, acquired by nefarious actors, and used for harm. Thus, any collaborative laboratory enterprise between the United States and other countries should have consensus-based policies and protocols in place to ensure that dual-use research findings and materials, including select agent pathogen strains, cannot be employed in these ways.

Beyond the study of high-containment pathogens, GOF research represents a special category of dual-use research of concern (DURC); in fact, a pause was implemented on influenza and coronavirus GOF from 2014 to 2017 (21) so that policymakers could determine how best to mitigate the inherent and derived risks posed by such efforts. During the moratorium, the National Institutes of Health (NIH) supported studies to quantify GOF risks in both biosafety and biosecurity contexts (22), including the development of a “what if” scenario for a coronavirus laboratory leak. The study concluded that the United States’ stringent biosafety protocols would render the risk of a coronavirus lab leak quite low, but the report also presaged that unless community mitigation was significantly robust, wild-type or an enhanced-transmissibility SARS-CoV could seed international outbreaks.

Hence, an important policy question is under what circumstances should the United States be performing GOF research in other countries where different or less stringent biosafety and biosecurity policy and protocols compromise the rigor of U.S. standards? U.S. policy emphasizes the use of risk-benefit analyses in GOF research to ensure that (i) risks are identified prior to the onset of research, (ii) the benefits of the research outweigh the risks, and (iii) identified risks can be effectively mitigated. Given these risk/benefit considerations, we ask whether such assessments were made in conjunction with GOF experiments pursued at the WIV. Were substitute methods for studying SARS considered in the WIV? Recent analyses suggest that the answer to these questions is no (23), and to further obfuscate the issue, the NIH has removed GOF information from its website (24) rather than elaborating details.

## WHAT STEPS TOWARD MODERNIZATION CAN, AND SHOULD, BE TAKEN?

We believe such inquiry, and the relative paucity of answers, suggest ways that biosecurity policies could be modernized post-COVID. National differences in biosafety and biosecurity standards render United States-centric GOF model scenarios less useful in the context of global bioevents like COVID-19. Therefore, we propose that U.S. GOF policies be revised to incorporate risk-benefit assessments as indisputable norms for GOF research in international settings.

Current U.S. policy on GOF research (25) explicates that risks-benefit analysis is an important factor in determining if the information sought could be acquired through other, less risky experimental protocols. Consistent with an emphasis upon the need to modernize biotechnology capabilities in response to bioevents (26), technological advances should also be considered for mitigating the risks associated with GOF research. For example, *in silico* machine learning algorithms may provide insights into which genetic mutations could lead to zoonotic spread to humans or species jumps that pose risks to livestock or agriculture. Such machine learning could supplant the need for wet bench creation of altered pathogens, as shown in promising early analyses of influenza (27). Moreover, such models have been initiated to study the current pandemic’s propensity for mutations (28). Of course, this would not entirely mitigate risk; GOF research databases could be vulnerable to hacking and their information subject to nefarious use (29). Still, such steps could minimize accidental release or laboratory-acquired infections by eliminating the need to create novel, living pathogenic organisms with gained traits that have adverse human or environmental/economic consequences.

The basic public health capabilities needed for a bioevent, whether natural or perpetrated, are generally the same. However, in the case of bioevents of indeterminate origin, and absent identification of “patient 0,” the additional tools of microbial forensics toward pathogen identification and attribution become critical. A recent study of all biosafety, biosecurity, and biodefense policies, governance, and executive branch directives identified the lack of programs in forensics and attribution as a fundamental gap in response capability (30). Despite federal efforts in support of microbial forensics following the 2001 anthrax attacks and the requirement for forensic tools to distinguish natural from perpetrated bioevents in the National Biodefense Strategy, the United States has not designated any specific microbial forensic tools standard for detecting engineered strains in a pandemic or other bioevent. Dedicated research programs such as FELIX and FunGCAT have developed promising proof-of-concept approaches to identify genetically engineered organisms and their laboratory of origin, and the increasing availability of such tools could fortify and benefit biosecurity by bridging the gap in attribution forensics (31).

Furthermore, such technologies and tools could be extended for use in the international arena in the event of suspected or alleged biological attacks. A qualified laboratory network focused on diagnostic analysis and forensic evidence has recently been convened (32) and is well positioned and equipped (with resources and personnel) to be effective in an international bioevent. We believe that the incorporation of state-of-the-science forensic and attribution methods would make this network even more capable and efficient.

While laboratory forensics are vital to *post facto* (i.e., “right-of-bang”) assessment and attribution of bioevents, oversight and governance of nations’ bioscience programs, inclusive of GOF and DURC, are important to *pre facto*, “left-of-bang” preparedness. Treaties that support global governance, including the Biological Weapons Convention (BWC), were established in the post-Cold War era to regulate the development or use of biological weapons in the broadest sense. However, the BWC was originally predicated on exemplars of prior bioweapons programs, existing pathogens, and their potential use as weapons of mass destruction and did not include investigatory capabilities for violations of the agreement (as noted in the Chemical Weapons Convention).

Therefore, as current biotechnological capabilities advance and expand the potential palette of agents that could incur a bioevent (33), and in the aftermath of the rapidity and extent of COVID’s global spread, there is renewed interest in revising the BWC to meet the risk and threat realities posed by radical leveling and emerging technologies. We are encouraged by the U.S. ambassador’s invocation at the Meeting of State Parties (34) that the BWC has been “treading water,” noting that COVID-19 should be a wakeup call to further operationalizing the BWC with consideration of ways that science and technology advances may pose risk and threat (35). At the recent COVID-19 Summit, the WHO proposed that a global health threat council, staffed by global leaders, be created (36). Such steps are warranted and needed at the global level. However, any call to action must ask sentinel questions that guide oversight and governance activities to their greatest benefit. a global health threat council could, for example, address GOF research in international settings and account for how LAIs should be appropriately reported and managed.

## CONCLUSIONS

While COVID’s exact origins may continue to be enigmatic, the pandemic crisis affords examination of laboratory biosafety and biosecurity procedures. “What if” scenarios can meaningfully contribute to assessment and revision of risk/assessment frameworks for GOF and DURC studies and can be employed to modernize preparedness and response programs as consistent with both scientific capability and the multinational sociopolitical environment(s) in which such research is conducted. Toward these goals, we propose and advocate the following recommendations:

• U.S. biosecurity and biosafety policies should be revisited to more thoroughly vet international laboratory funding for GOF and DURC;
• the United States should revitalize biosafety and biosecurity advisory committees such as the National Science Advisory Board for Biosecurity (NSABB) to collaborate with international allies, and peer competitors, in oversight and regulation of GOF and DURC activities;
• the United States should select and invest in standards for forensic analytical tools and methods that can be routinely applied in response to bioevents; these approaches should be explicated in biodefense strategies and shared internationally;
• the United States should invest in research to develop alternate means for studying GOF that minimizes the chances of future outbreaks and should continue to support rigorous risk-benefit assessments of GOF and DURC;
• international fora should evaluate the merits of a multinational system of norms and standards for more effective and efficient monitoring and oversight of the increasing number of high containment laboratories worldwide, including routine reporting of LAIs.

These recommendations are broad in scope and will require diligent (economic and political) support. Such effort and investment represent important and necessary elements to enable continuing biological research, inclusive of GOF and DURC activities, in ways that are commensurate with current scientific and technological capability, focal to their benefits and benevolent intent, and concomitantly cognizant of, and ever prepared for, extant and emerging risks and threats.
